# Interleukin-24 regulates mucosal remodeling in inflammatory bowel diseases

**DOI:** 10.1186/s12967-021-02890-7

**Published:** 2021-06-02

**Authors:** Anna Ónody, Apor Veres-Székely, Domonkos Pap, Réka Rokonay, Beáta Szebeni, Erna Sziksz, Franz Oswald, Gábor Veres, Áron Cseh, Attila J. Szabó, Ádám Vannay

**Affiliations:** 1 1st Department of Pediatrics, Semmelweis University, Budapest, Hungary; 2 ELKH-SE Pediatrics and Nephrology Research Group, 53-54 Bókay J. Street, Budapest, H-1083 Hungary; 3 Department of Internal Medicine I, University Medical Center, Ulm, Germany; 4 Pediatric Institute-Clinic, University of Debrecen, Debrecen, Hungary

**Keywords:** Inflammatory bowel diseases, IBD, Interleukin, IL-24, Tissue remodeling, Fibrosis

## Abstract

**Background:**

Recently, increased interleukin (IL)-24 expression has been demonstrated in the colon biopsies of adult patients with inflammatory bowel disease (IBD). However, the role of IL-24 in the pathomechanism of IBD is still largely unknown.

**Methods:**

Presence of IL-24 was determined in the samples of children with IBD and in the colon of dextran sodium sulfate (DSS) treated mice. Effect of inflammatory factors on *IL24* expression was determined in peripheral blood (PBMCs) and lamina propria mononuclear cells (LPMCs). Also, the impact of IL-24 was investigated on HT-29 epithelial cells and CCD-18Co colon fibroblasts. Expression of tissue remodeling related genes was investigated in the colon of wild type (WT) mice locally treated with IL-24 and in the colon of DSS treated WT and *Il20rb* knock out (KO) mice.

**Results:**

Increased amount of IL-24 was demonstrated in the serum and colon samples of children with IBD and DSS treated mice compared to that of controls. IL-1β, LPS or H_2_O_2_ treatment increased the expression of *IL24* in PBMCs and LPMCs. IL-24 treatment resulted in increased amount of TGF-β and PDGF-B in HT-29 cells and enhanced the expression of extracellular matrix (ECM)-related genes and the motility of CCD-18Co cells. Similarly, local IL-24 treatment increased the colonic *Tgfb1* and *Pdgfb* expression of WT mice. Moreover, expression of pro-fibrotic *Tgfb1* and *Pdgfb* were lower in the colon of DSS treated *Il20rb* KO compared to that of WT mice. The disease activity index of colitis was less severe in DSS treated *Il20rb* KO compared to WT mice.

**Conclusion:**

Our study suggest that IL-24 may play a significant role in the mucosal remodeling of patients with IBD by promoting pro-fibrotic processes.

**Supplementary Information:**

The online version contains supplementary material available at 10.1186/s12967-021-02890-7.

## Background

Incidence of inflammatory bowel disease (IBD) including Crohn’s disease (CD) and ulcerative colitis (UC) has almost doubled during the last two decades, indicating its growing significance [1, 2]. IBD is characterized by inappropriate ongoing activation of immune responses against environmental factors in genetically susceptible individuals [3, 4].

As a consequence of chronic intestinal inflammation multitude of cytokines and growth factors are released contributing to intense mucosal remodeling. In this process, subepithelial myofibroblasts (MFs) play a determinative role. Under physiological conditions, MFs participate in the maintenance of the basement membrane underlying the epithelial layer, thereby supporting mucosal integrity [5]. However, chronic inflammation in IBD may lead to permanent activation of MFs shifting the processes of tissue repair toward the excessive deposition of extracellular matrix (ECM). Finally, increased submucosal accumulation of connective tissue lead to intestinal motility disorders, malabsorption and in severe cases critical narrowing of the lumen [6]. Indeed, approximately half of the IBD patients are severely affected and need one or more surgical intervention over their lifetime [5, 7].

Interleukin (IL)-20 subfamily contains five related molecules, of which IL-19, IL-20 and IL-24 act on IL-20RA/IL-20RB and on IL-22RA/IL-20RB common receptor heterodimers [8]. These cytokines are mainly produced by leukocytes [9, 10] and also by other cell types including epithelial cells and MFs [11, 12]. The most well-known function of the subfamily is the regulation of innate immune responses [13–15], however recently, we and others suggested its importance in the process of tissue repair and fibrosis of different organs [16–18]. Previously, increased presence of IL-24 was demonstrated in the intestinal biopsies of patients with IBD and coeliac disease [19–21]. Although it has been suggested that IL-24 protects the integrity of the mucosal epithelial layer [19, 21] the exact biological role of IL-24 in IBD is still largely unknown.

In the present study we aimed to investigate the possible role of IL-24 in repair process of the chronically inflamed intestinal mucosa of patients with IBD. Therefore, we investigated the expression of IL-24 in the colon mucosa of children with IBD and also in the colon of dextran sodium sulfate (DSS) treated mice. In vitro experiments were performed to determine the effect of different inflammatory factors on IL-24 expression by PBMCs. In vitro and in vivo experiments using HT-29 or CCD-18Co cells and wild type (WT) and *Il20rb* knock out (KO) mice were performed to investigate the effect of IL-24 on the process of intestinal remodeling.

## Methods

### Patients

Colonic biopsies and serum samples from pediatric patients were obtained at the 1^st^ Department of Pediatrics, Semmelweis University, Hungary. The study was approved by the Semmelweis University Regional and Institutional Committee of Science and Research Ethics (TUKEB 175/2007).

IBD was diagnosed according to “The Porto criteria” [22, 23], activity score was evaluated according to the Pediatric Crohn’s Disease Activity Index (PCDAI) and Pediatric Ulcerative Colitis Activity Index (PUCAI) [24, 25]. All of enrolled patients with IBD were newly diagnosed and untreated at the time of sampling. In case of the control children, as a part of gastroenterological examination blood sampling and colonoscopy was carried out on the grounds of chronic abdominal pain, diarrhea or polyposis of the colon. During the diagnostic procedure IBD was excluded, colonic biopsy specimens showed normal appearance and histology in these cases. Clinical characteristics of patients enrolled into colonic tissue and serum analyses are described below.

#### Colonic biopsy samples

For Western blot analysis, biopsy samples derived from colon transversum of the children were taken from controls (n = 10), macroscopically inflamed (n = 7) and non-inflamed regions (n = 7) of the mucosa in children with CD and from the inflamed mucosa of UC patients (n = 7). Clinical characteristics of pediatric patients are described in Table 1. Biopsy samples were immediately snap-frozen and stored at − 80 °C until further analysis.

**Table 1:** Clinical characteristics of pediatric patients at colonic biopsy

	Control	CD (non-inflamed)	CD (inflamed)	UC
Number of patients	10	7	7	7
Gender	3M/7F	4M/3F	4M/3F	3M/4F
Age (year)	12.76 ± 4.15	13.11 ± 1.65	13.74 ± 2.29	14.52 ± 1.40
CRP (mg/l)	0.87 ± 1.37	30.15 ± 28.94	26.48 ± 31.03	7.43 ± 10.30
PCDAI/PUCAI	0	28.78 ± 10.51	27.50 ± 16.96	31.00 ± 20.12

Results are presented as mean ± SD. *CD* Crohn’s disease, *UC* colitis ulcerosa, *M* male, *F* female, *CRP* C-reactive protein, *PCDAI* Pediatric Crohn’s Disease Activity Index, *PUCAI* Pediatric Ulcerative Colitis Activity Index

#### Serum samples

For ELISA, blood samples were taken from controls (n = 15) and from children with CD (n = 12). The blood samples were centrifuged, and the resulting serum samples were immediately frozen and stored at − 80 °C until further analysis. Clinical characteristics of patients are described in Table 2.

**Table 2:** Clinical characteristics of pediatric patients at serum collection

	Control	CD
Number of patients	15	12
Gender	8M/7F	6M/6F
Age (year)	5.8 ± 4.42	12.31 ± 5.657
CRP (mg/l)	3.615 ± 11.54	45.08 ± 40.63
PCDAI	0	27.75 ± 13.82

Results are presented as mean ± SD. *CD* Crohn’s disease, *M* male, *F* female, *CRP* C-reactive protein, *PCDAI* Pediatric Crohn’s Disease Activity Index, *PUCAI* Pediatric Ulcerative Colitis Activity Index

### Animals and treatment protocol

All experiments were approved by the institutional committee on animal welfare (PEI/OO1/83–4/2013). Animals were housed in a temperature-controlled (22 ± 1 °C) room with alternating light and dark cycles and had free access to standard chow and water.

#### Intracolonic injection

Experiments were performed on 7–8-week-old male WT C57BL/6 J (Charles River Laboratories, Sulzfeld, Germany) mice. Mice were randomized into two groups (control and IL-24 treated; n = 6/groups). After general anesthesia by inhalation of isoflurane (3% V/V) mixed with air using a vaporizer (Eickemeyer Veterinary Equipment Ltd., Twickenham, UK) and standard midline laparotomy the bowel was gently displaced from the abdomen. Intracolonic treatment with IL-24 was performed based on the method previously described by Boni et al. [26]. Briefly, recombinant IL-24 (0.1 µg/50 µl/mouse; R&D Systems, Minneapolis, MN, USA) was intraparietally injected into the antimesenteric side of the distal colon of each animal at a distance of 30 mm from the anus. Control animals were similarly treated with vehicle only (phosphate buffered saline, PBS, 50 µl/mouse). Mice were harvested 24 h after the onset of injection and treated colon segments were surgically removed, snap-frozen and stored at − 80 °C until further analysis.

#### DSS-induced colitis

In order to induce colitis, 7–8 week old male *Il20rb* KO (the genetic background of the mice was C57BL/6 J, obtained from Franz Oswald, University Medical Center, Ulm, Germany) [27] and corresponding WT mice were treated with dextran sulfate sodium salt (DSS) dissolved in their drinking water (2.5% (w/v), MP Biomedicals, LLC, Santa Ana, CA, USA; n = 6/groups) for 7 days. Thereafter mice were watered with normal water for another 12 days. Control animals (WT control and *Il20rb* KO control; n = 6/groups) received normal drinking water only. Proximal colon sections of WT and *Il20rb* KO mice were surgically removed on the 19th day (WT DSS; *Il20rb* KO DSS, n = 6/groups) after the beginning of their DSS treatment. Bowel segments were immediately snap frozen and stored at − 80 °C until further analysis or fixed in formalin (4%, pH = 7.4).

Clinical parameters, including body weight, stool consistency and blood content, and general conditions, behavioral indicators of abdominal pain were monitored daily during the whole experiment. Disease activity index (DAI) was calculated by summing of well-established and validated scores for parameters (described in Table 3), that are somewhat analogous to the clinical presentation of human IBD [28]. Body weight change and DAI in different groups were compared by daily values, and area under curve (AUC) of the given parameter of individual mice, as well.

**Table 3:** Determination method of disease activity index (DAI) in DSS-induced colitis model of mice

Partial score	Stool consistency	Bleeding	General conditions
0	Normal	None	Normal
1	Soft pellets	Blood spots in stool	Hunchbacked, piloerection
2	Formed, but thin pellets	Deep red stool, perianal bleeding	Hunchbacked, piloerection, reduced locomotor activity
3	Liquid, diarrhea	Gross bleeding	–

DAI was determined individually determined in each day of the experiment by summing partial scores of disease symptoms

### Cell lines

#### HT-29 colon epithelial cell culture

HT-29 human colon epithelial cell line (Sigma-Aldrich Co., St. Louis, MO, USA; European Collection of Cell Cultures) was cultured in modified McCoy’s 5A medium (Sigma-Aldrich) supplemented with L-glutamine, 10% heat-inactivated fetal bovine serum (FBS) (Invitrogen, Carlsbad, CA, USA) 1% streptomycin and penicillin (Sigma-Aldrich) at 37 °C and 5% CO_2_. To perform mRNA expression analysis and Annexin/PI staining, HT-29 cells were seeded in 6-well plates (n = 6 well/treatment group) at a density of 5 × 10^5^ cells/well After plating, cells were treated with recombinant IL-24 (100 ng/ml, R&D). Cells were subsequently incubated for 24 h in 37 °C. Control cells were treated with vehicle only.

#### CCD-18Co colon fibroblast cell culture

CCD-18Co human colon fibroblast cell line (ATCC, Manassas, VA, USA) was cultured in Eagle's Minimum Essential Medium (EMEM) (ATCC) supplemented with L-glutamine, 10% heat-inactivated FBS (Invitrogen) and 1% streptomycin and penicillin (Sigma-Aldrich) at 37 °C and 5% CO_2_. To perform mRNA expression analysis and Annexin/PI staining, CCD-18Co cells were seeded in 6-well plates (n = 6 well/treatment group) at a density of 5 × 10^5^ cells/well, for fibroblast migration, MTT and SiriusRed assays, cells were seeded into 96-well plates at a density of 10^4^ cells/well (n = 5 well/treatment group). After plating, cells were treated with recombinant IL-24 (100 ng/ml, R&D), TGF-β1 (1 nM, R&D) or PDGF-B (10 ng/ml, R&D). Control cells were treated with corresponding solvents (IL-24 and PDGF-B: PBS; TGF-β1: 4 mM HCl) alone.

#### Peripheral blood mononuclear cells (PBMCs)

PBMCs from control pediatric patient (5 yearsoldmale, gastroenterological examination due to tongue plaque andhalitosis) were isolated by density gradient centrifugation using Histopaque-1077 (Sigma-Aldrich). After isolation, cells were placed into RPMI 1640 medium (ATCC) supplemented with L-glutamine, 10% FBS and 1% streptomycin and penicillin mixture. To perform mRNA expression analysis, PBMCs were seeded into 24-well plates at a density of 5 × 10^5^ cells/well (n = 6 well/treatment group) and treated either with recombinant IL-1β (100 ng/ml; Invitrogen), LPS (100 ng/ml; Lipopolysaccharides from Escherichia coli, Sigma-Aldrich), recombinant TNF-α (10 ng/ml; R&D), recombinant TGF-β1 (0.5 nM; Invitrogen), recombinant IL-17 (100 ng/ml; R&D), or 25 µM H_2_O_2_ for 24 h. Vehicle treated cells served as controls.

#### Lamina propria mononuclear cells (LPMCs)

LPMCs were isolated from colon tissue of a control WT mouse based on the method described by McManus et al. [29]. Briefly, the total colon was harvested and cleaned thoroughly by PBS, thereafter cut into small pieces. Tissue fragments were digested in collagenase containing solution (1 mg/ml; Sigma-Aldrich), then mechanically disaggregated and filtered using Falcon 40 μm cell strainer (Thermo Fisher Scientific, Waltham, MA, USA) to obtain single-cell suspension. LPMCs were then isolated by density gradient centrifugation using Histopaque-1077 (Sigma-Aldrich). After isolation, cells were placed into RPMI 1640 medium (ATCC) supplemented with L-glutamine, 10% FBS and 1% streptomycin and penicillin mixture. To perform PCRs LPMCs were seeded into 96-well plates at a density of 5 × 10^5^ cells/well (n = 6 well/treatment group) and treated either with recombinant IL-1β (100 ng/ml; Invitrogen), LPS (100 ng/ml; Lipopolysaccharides from Escherichia coli, Sigma-Aldrich), recombinant TNF-α (10 ng/ml; R&D), recombinant TGF-β1 (0.5 nM; Invitrogen), recombinant IL-17 (100 ng/ml; R&D), or 25 µM H_2_O_2_ for 24 h. Vehicle treated cells served as controls.

### Annexin V/propidium iodide staining

Apoptosis assay was performed using FITC Annexin V Apoptosis Detection Kit I (BD Pharmingen, San Jose, CA, USA) according to the manufacturer’s recommendations. Flow cytometry analysis was performed using a FACS Aria cytometer (BD). Cells negative for Annexin V and PI were referred as viable cells, cells positive for Annexin V and negative for PI were referred as early apoptotic cells and cells positive for Annexin V and PI were referred as late apoptotic cells. Necrotic cell debris were negative for Annexin V and positive for PI.

### Fibroblast migration assay

CCD-18Co cells were seeded into 96 well-plates containing non-toxic gel barriers creating cell-free zones. After 24 h of incubation, barriers were removed, and the medium was completed with recombinant IL-24 or with vehicle only. Bright-field images of each well were taken using Olympus IX81 microscope system (Olympus Corporation, Tokyo, Japan) right after treatment (pre-migration) and 5 days after treatment (post-migration). Cell-free gap area was measured using ImageJ 1.48v software (The National Institutes of Health, Bethesda, MD, USA) and determined as a ratio of initial gap area at 0 h.

### MTT cell proliferation assay

MTT cell proliferation and viability assay was performed by using Cell Proliferation Kit I (Roche Diagnostics, Mannheim, Germany) according to the manufacturer’s recommendations. Absorbance was recorded at 570 nm and at 690 nm as background using a Hidex Chameleon Microplate Reader (Triathler, Plate Chameleion, 300SL Lablogic Systems Inc., Brandon, FL, USA) using MikroWin 2000 software.

### SiriusRed collagen detection assay

Reagents for in vitro SiriusRed collagen detection assay [30] were purchased from Sigma-Aldrich. After fixation (26% EtOH, 3.7% formaldehyde, 2% glacial acetic acid), plates were incubated for 15 min at room temperature (RT). Then cells were stained for 1 h with 0.1% solution of SiriusRed (DirectRed80) dissolved in 1.2% picric acid and washed with 0.1 M HCl. To elute collagen bounded dye, 100 µl 0.1 M NaOH was added and optical density was determined at 544 nm and at 690 nm as background using Hidex Chameleon Microplate Reader with MikroWin 2000 software.

### LDH cytotoxicity assay

LDH assay was performed as previously described [31]. All reagents were purchased from Sigma-Aldrich. Absorbance was recorded at 570 nm and at 690 nm as background in a Hidex Chameleon Microplate Reader using MikroWin 2000 software.

### Immunofluorescence staining

Human colon biopsies and mice bowel samples were embedded into Shandon cryomatrix (Thermo Fisher Scientific) and cut into 5 μm slides, stored at -80 °C until use. HT-29 and CCD-18Co cells were seeded in chambers and cultured for 24 h in 37 °C. After repeated washing slides were permeabilized with Cytofix/Cytoperm (BD) for 15 min at RT, then incubated with primary antibodies specific to αSMA (sc-53015; mouse, 1:2000, Santa Cruz Biotechnology, Dallas, TX, USA) or IL-20RB (ab124332; rabbit, 1:100, Abcam) for 1 h at RT. After repeated washing slides were incubated with Alexa Fluor 488 goat anti-mouse IgG (A11001, Invitrogen) and Alexa Fluor 568 donkey anti-rabbit secondary antibody (A10042, Invitrogen), both diluted to 1:100 for 30 min at RT in the dark and counterstained with Hoechst 33,342 (1:2000, Sigma-Aldrich). Finally, slides were rinsed in PBS and coverslipped with Vectashield fluorescent mounting medium (Vector Laboratories, Burlingame, CA, USA). Appropriate controls were performed omitting the primary antibodies to assure their specificity and to avoid autofluorescence. Sections were analyzed with a Nikon C2 confocal laser scanning microscope system (Nikon, Minato, Tokyo, Japan).

### RNA isolation and cDNA synthesis

Total RNA was isolated from mouse colon tissue samples, HT-29 and CCD-18Co cells by Total RNA Mini Kit (Geneaid Biotech Ltd., New Taipei City, Taiwan) according to the instructions of the manufacturer. Concentration and quality of the isolated RNA were determined by DeNovix DS-11 spectrophotometer (DeNovix Inc., Wilmington, DE, USA). A total of 500 ng RNA was reverse-transcribed using Maxima First Strand cDNA Synthesis Kit for RT-qPCR (Thermo Fisher Scientific) to generate first-stranded cDNA.

### Real-time polymerase chain reaction (PCR)

Expression of the examined target genes was measured by real-time PCR on a Light Cycler 480 system (Roche). Nucleotide sequences of the applied primer pairs, their specific optimal annealing temperatures and product lengths are shown in Additional file 1. Results were analyzed by Light-Cycler 480 software version 1.5.0.39 (Roche). Relative mRNA expressions were determined by comparison with *GAPDH* as internal control using the ∆∆Ct method [32]. Data were normalized and presented as the ratio of their control values.

### Enzyme-linked immunosorbent assay (ELISA)

ELISA analysis of IL-24 from serum was performed using Cloud Clone Corporation ELISA Kit for Interleukin 24 (Cloud-Clone Corp., Houston, Texas, USA) according to the manufacturer. Absorbance was measured at 450 nm using Hidex Chameleon Microplate Reader and MikroWin 2000 software. Concentration of IL-24 in the samples was determined by comparing the absorbance of samples to the standard curve.

### Flow cytometry

HT-29 cells were centrifuged, washed with PBS and incubated for 10 min at RT with FACS Permeabilizing Solution 2 (BD). Permeabilized cells were washed with PBS and incubated with primary anti TGF-ß1 (sc-146, rabbit, Santa Cruz) or anti PDGF-B (sc-7878, rabbit, Santa Cruz) antibody diluted to 1:50 for 30 min at RT. Cells were subsequently washed with Permeabilizing Solution 2 and incubated with Alexa Fluor 568 donkey anti-rabbit secondary antibody (A10042, Invitrogen) diluted to 1:200 for 30 min at RT in the dark. Negative controls were incubated with the secondary antibody alone. The flow cytometric analysis was carried out using a FACSAria cytometer (BD). The mean fluorescence intensity (MFI) values of each sample were normalized and presented as the ratio of their control values.

### Western blot analysis

Tissue samples were homogenized in lysis buffer containing 50 mM HEPES, 150 mM NaCl, 1% Triton X-100, 5 mM EDTA, 5 mM EGTA, 20 mM sodium pyrophosphate, 20 mM NaF, 0.2 mg/mL phenylmethylsulfonyl fluoride, 0.01 mg/mL leupeptin, and 0.01 mg/mL aprotinin (pH 7.4; each substance was obtained from Sigma-Aldrich). Protein concentration was determined in triplicates by a detergent-compatible protein assay (Bio-Rad, Hercules, CA). Denatured samples (20 μg protein/lane) were loaded and separated on 4–20% gradient SDS polyacrylamide gel, and transferred to nitrocellulose membranes. To verify the transfer, membranes were stained with Ponceau S (Sigma Aldrich), then washed and blocked with 5% non-fat milk in TRIS-buffered saline (TBS) for 1 h at RT. Thereafter the membranes were incubated overnight at 4 °C with antibodies specific for IL-24 (ab182567; 1:1000, Abcam, Cambridge, United Kingdom), αSMA (sc-53015; 1:10,000, Santa Cruz), fibronectin (FN) (ab2413; 1:2000, Abcam), pro-collagen type I alpha 2 (pro-COL1A2) (sc-166572; 1:2000. Santa Cruz) or GAPDH (sc-47724; 1:2000, Santa Cruz). After repeated washing with TBS containing 0.05% Tween-20 and 1% non-fat milk, membranes were incubated with the corresponding HRP-conjugated secondary antibodies (1:2000 anti-rabbit or anti-mouse, Santa Cruz) for 1 h at RT. Bands of interest were detected using enhanced chemiluminescence detection (Western Blotting Luminol Reagent, GE Healthcare, Waukesha, WI) and quantified by densitometry (VersaDoc, Quantity One Analysis software; Bio-Rad) as integrated optical density after subtraction of background. Relative protein levels were determined by comparison with GAPDH as internal control. Data were normalized and presented as the ratio of control values.

### Statistical analysis

Statistical evaluation and data were performed by GraphPad Prism 6.01 software (GraphPad Software Inc., La Jolla, CA, USA). After testing normality with Kolmogorov–Smirnov test, row data were analyzed with Mann–Whitney U-test or Kruskal–Wallis test to determine differences between corresponding groups. Multiple comparisons of row data derived from MTT, LDH, migration and SiriusRed assays were performed using multiple t-test and ordinary two-way ANOVA with Dunnett correction. p ≤ 0.05 was considered as statistically significant. Values were expressed as mean + SD.

## Results

### Presence of IL-24 and its receptor in pediatric patients with IBD

**Fig. 1:**
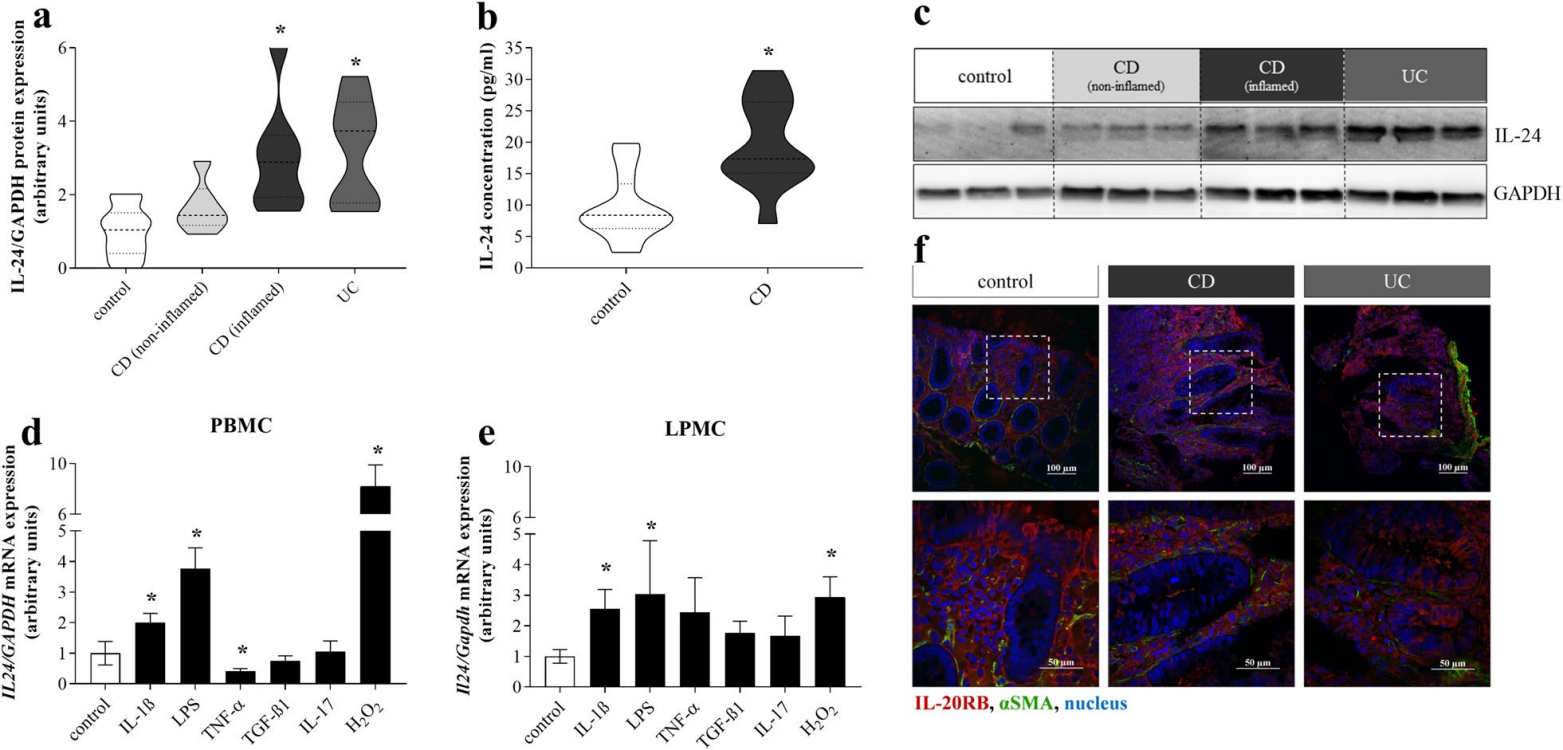
Presence of IL-24 and IL-20RB in IBD. Protein level of IL-24 in the colonic mucosa (n = 7–10) of children with CD, UC and controls was determined by Western blot analysis in comparison with GAPDH as internal control (**a**, **c**). Concentration of IL-24 in the serum (n = 12–15) of controls and children with CD was measured by ELISA (**b**). Violin plots indicate the distribution of IL-24 levels in the given groups. Relative mRNA expression of *IL24* in PBMCs (**d**) and LPMCs (**e**) after treatment with IL-1ß, LPS, TNF-α, TGF-ß1, IL-17 and H_2_O_2_ (n = 6) was measured by real-time PCR in comparison with *GAPDH* as internal control. Results are presented as mean ± SD. *p < 0.05 vs. control (Kruskal–Wallis test (**a**, **d**, **e**) or Mann–Whitney *U*-test (**b**)). Localization of IL-20RB (red) and αSMA (green) was determined by immunofluorescence staining of the colonic mucosa of controls and children with IBD (**f**). Nuclei are stained with Hoechst33342 (blue). Scale bars: 100 µm and 50 µm

First, we analyzed the presence of IL-24 in human colon biopsies and serum samples. Protein level of IL-24 was increased in inflamed mucosa of pediatric patients with CD and UC compared to controls, but it was unchanged in the non-inflamed colonic region of patients with CD (Fig. 1a, c). Serum IL-24 level was also elevated in CD group compared to controls (Fig. 1b).

Moreover, immunofluorescence staining was performed to determine the colonic localization of IL-20RB receptor subunit. We observed IL-20RB immunoreactivity in colonic lamina propria, basal region of epithelial cells and subepithelial MFs in control, CD and UC samples, as well (Fig. 1f; representative images of a control patient (12 years old female with colon poly p), a pediatric patient with newly diagnosed CD (17 years old male, PCDAI: 65) and UC (8 years old male, PUCAI: 20)).

### Presence of IL-24 in mice with DSS-induced colitis

**Fig. 2:**
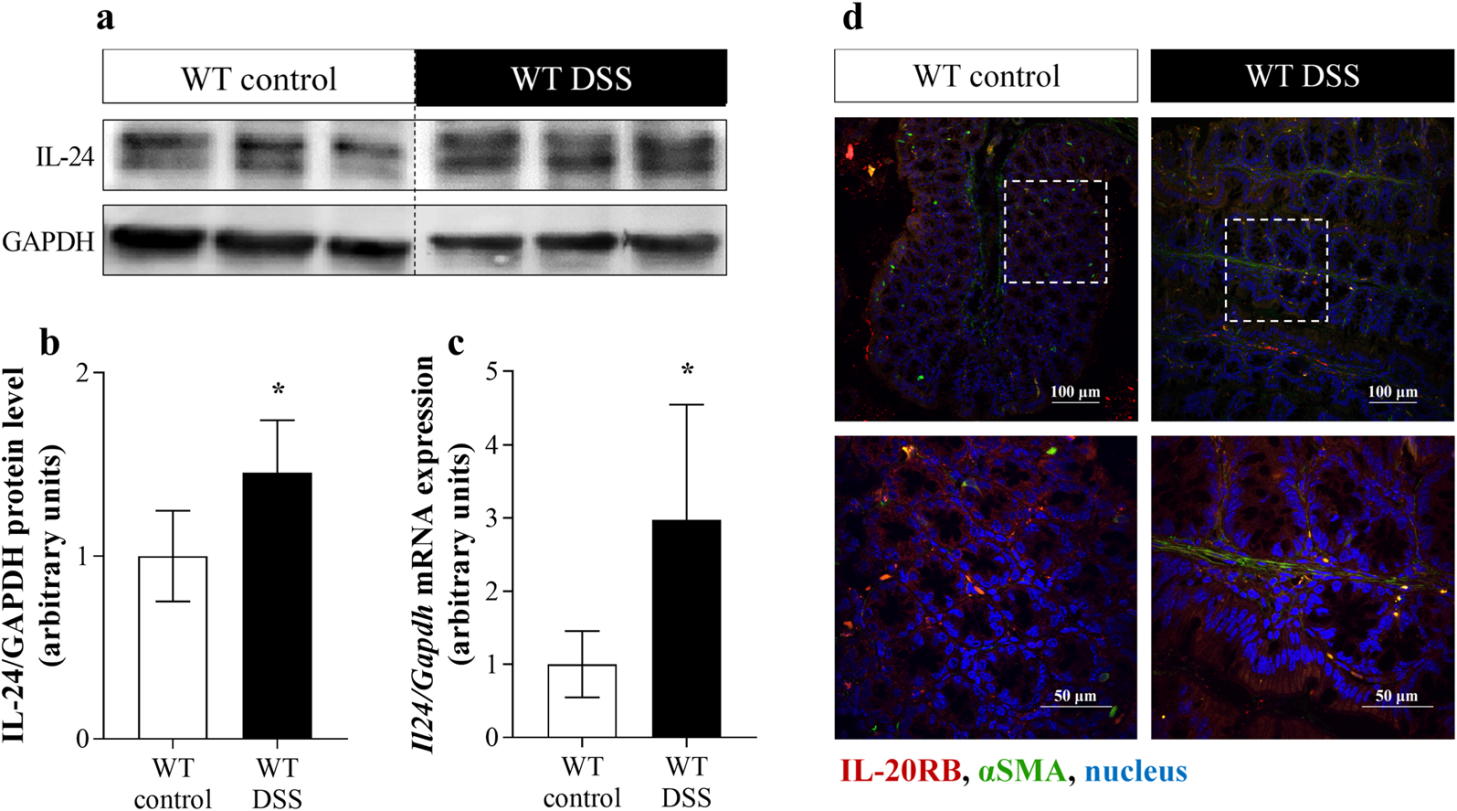
Presence of IL-24 and IL-20RB in WT mice with DSS-induced colitis. Protein level and mRNA expression of IL-24 in colon tissue (n = 6) was determined by Western blot analysis (**a**, **b**), and real-time PCR (**c**) by comparison with GAPDH as internal control, respectively. Results are presented as mean ± SD. *p < 0.05 vs. WT control (Mann–Whitney *U*-test). Localization of IL-20RB (red) in the colonic tissue samples from control and DSS-treated WT mice was determined by immunofluorescence staining with αSMA (green) co-localization (**d**). Nuclei are stained with Hoechst33342 (blue). Scale bars: 100 µm and 50 µm

To further study the role of IL-24 in intestinal pathophysiology, we investigated its expression in the colon of mice with DSS induced colitis. DSS treatment of WT mice increased the protein level (Fig. 2a, b) and also the mRNA expression (Fig. 2c) of *Il24* in the colon. Moreover, strong IL-20RB immunoreactivity was observed in lamina propria, crypt epithelial cells and subepithelial MFs of DSS-treated and control mice, as well (Fig. 2d).

### Effect of inflammatory factors on the IL-24 synthesis by PBMCs and LPMCs

To understand the mechanism leading to increased production of IL-24 the effect of IBD-related inflammatory factors on the IL-24 expression by PBMCs and LPMCs was investigated (Fig. 1d, e). While IL-1ß, LPS or H_2_O_2_ treatment of PBMCs or LPMCs increased the mRNA expression of *IL24*, TNF-α decreased *IL24* expression by PBMCs compared to vehicle treated control cells. TGF-ß1 or IL-17 treatment did not alter the *IL24* expression in PBMCs (Fig. 1d) or LPMCs (Fig. 1e).

### Effect of IL-24 on HT-29 colon epithelial cells

**Fig. 3:**
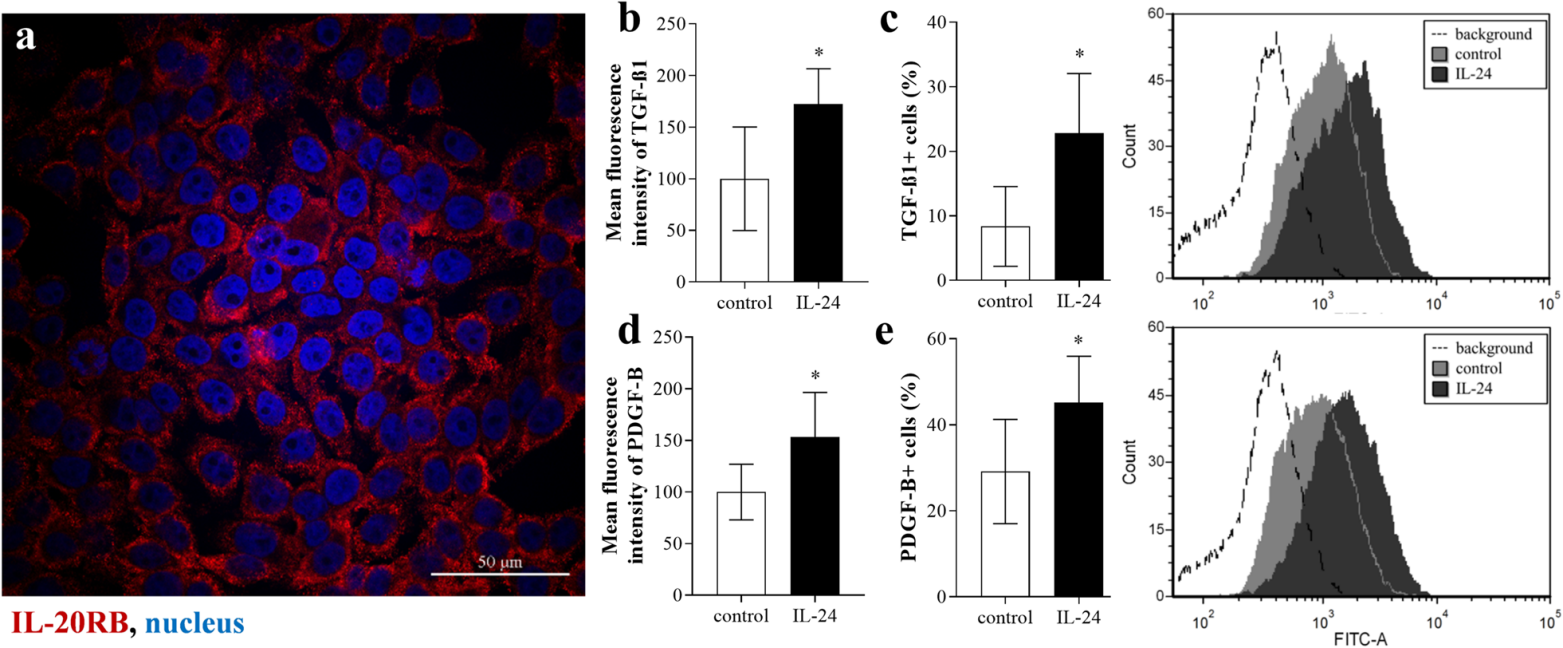
Effect of IL-24 on HT-29 colonic epithelial cells. Presence of IL-20RB (red) on HT-29 colon epithelial cells was determined by immunofluorescence staining (**a**). Nuclei are stained with Hoechst33342 (blue). Scale bar: 50 µm. After treatment with IL-24, mean fluorescence intensity of TGF-β1 (**b**) and PDGF-B (**d**), and percentage of TGF-β1 + (**c**) and PDGF-B + (**e**) cells was determined by flow cytometry (n = 6). Results are presented as mean ± SD. *p < 0.05 vs. control (Mann–Whitney U-test)

Similarly, to the epithelial cells of human colon biopsies or that of DSS treated mice IL-20RB immunoreactivity was observed on the HT-29 cells (Fig. 3a). IL-24 treatment did not alter viability of HT-29 cells (Additional file 2), but increased the mean fluorescence intensity of TGF-β1 (Fig. 3b) and PDGF-B (Fig. 3d) and also the percentage of TGF-β1 + (Fig. 3c) and PDGF-B + (Fig. 3e) cells.

### Effect of IL-24 on CCD-18Co colon fibroblast cells

**Fig. 4:**
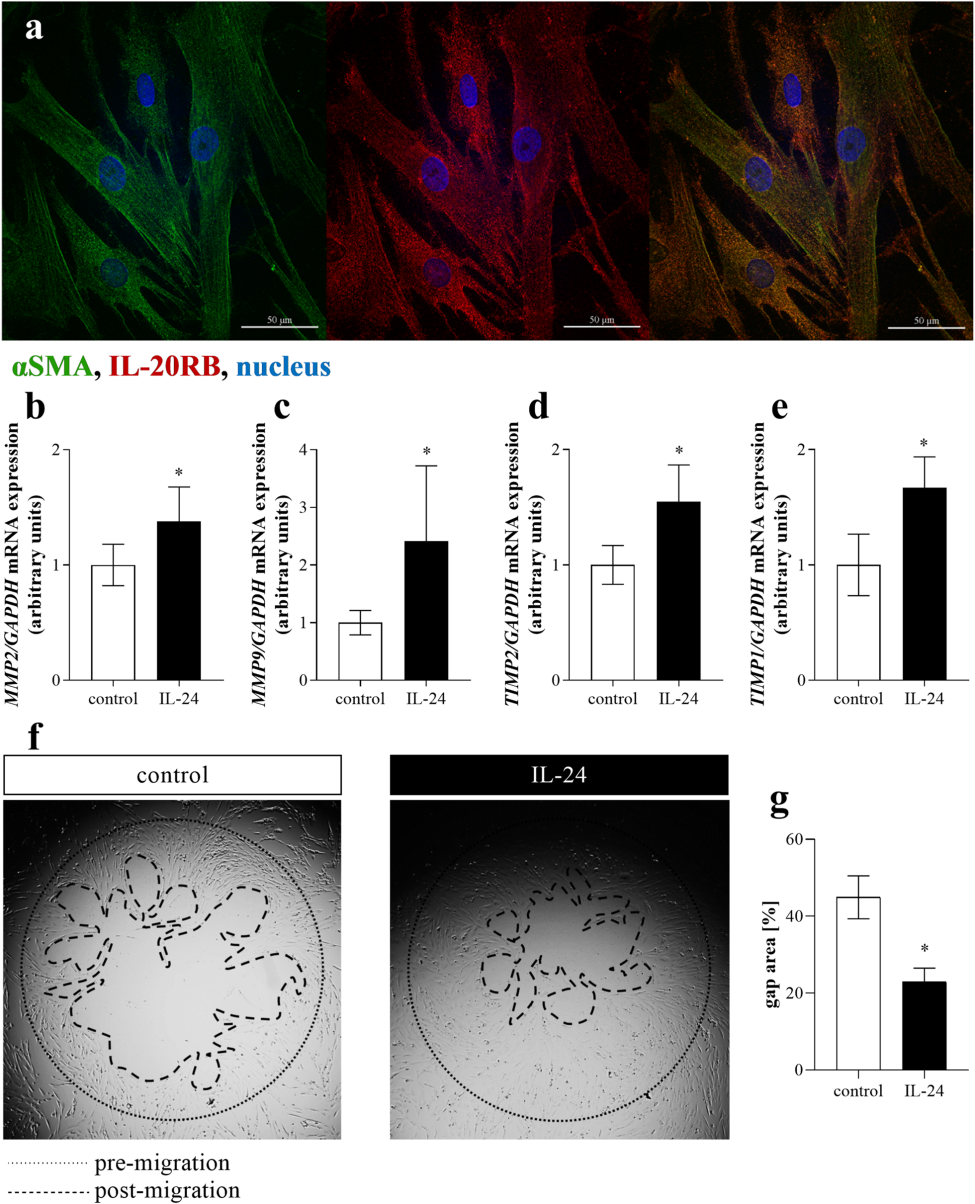
Effect of IL-24 on CCD-18Co cells. Presence of IL-20RB (red) and αSMA (green) on CCD-18Co colon fibroblast cells was determined by immunofluorescence staining (**a**). Nuclei are stained with Hoechst33342 (blue). Scale bar: 50 µm. The mRNA expression of *MMP2* (**b**), *MMP9* (**c**), *TIMP1* (**d**) and *TIMP2* (**e**) of CCD-18Co colon fibroblasts was determined by real-time PCR, by comparison with *GAPDH* as internal control (n = 6). Migration ability of CCD-18Co cells was investigated using fibroblast migration assay (**f**). The area of cell-free gap was measured after IL-24 treatment (post-migration) and determined as the ration of initial gap size (pre-migration) (**g**). Results are presented as mean ± SD. *p < 0.05 vs. control (Mann–Whitney *U*-test)

In accordance with our results demonstrating the presence of IL-20RB on the subepithelial MFs of human colon biopsies or on colon tissue of DSS treated mice we observed IL-20RB immunoreactivity on CCD-18Co colon fibroblast cells (Fig. 4a).

IL-24 treatment did not affect viability, proliferation (Additional file 3) or overall ECM production of CCD-18Co cells (Additional file 4). However, IL-24 treatment induced the mRNA expression of *COL3A1, FN1, MMP2*, *MMP9*, *TIMP1* and *TIMP2* by CCD-18Co cells compared to controls (Fig. 4b–e), and also enhanced the migration of CCD-18Co cells as demonstrated by the increased cell-free gap closure of the fibroblasts (Fig. 4e, f).

### Tissue remodeling-associated factors in colon tissue of WT mice after local treatment with IL-24

**Fig. 5:**
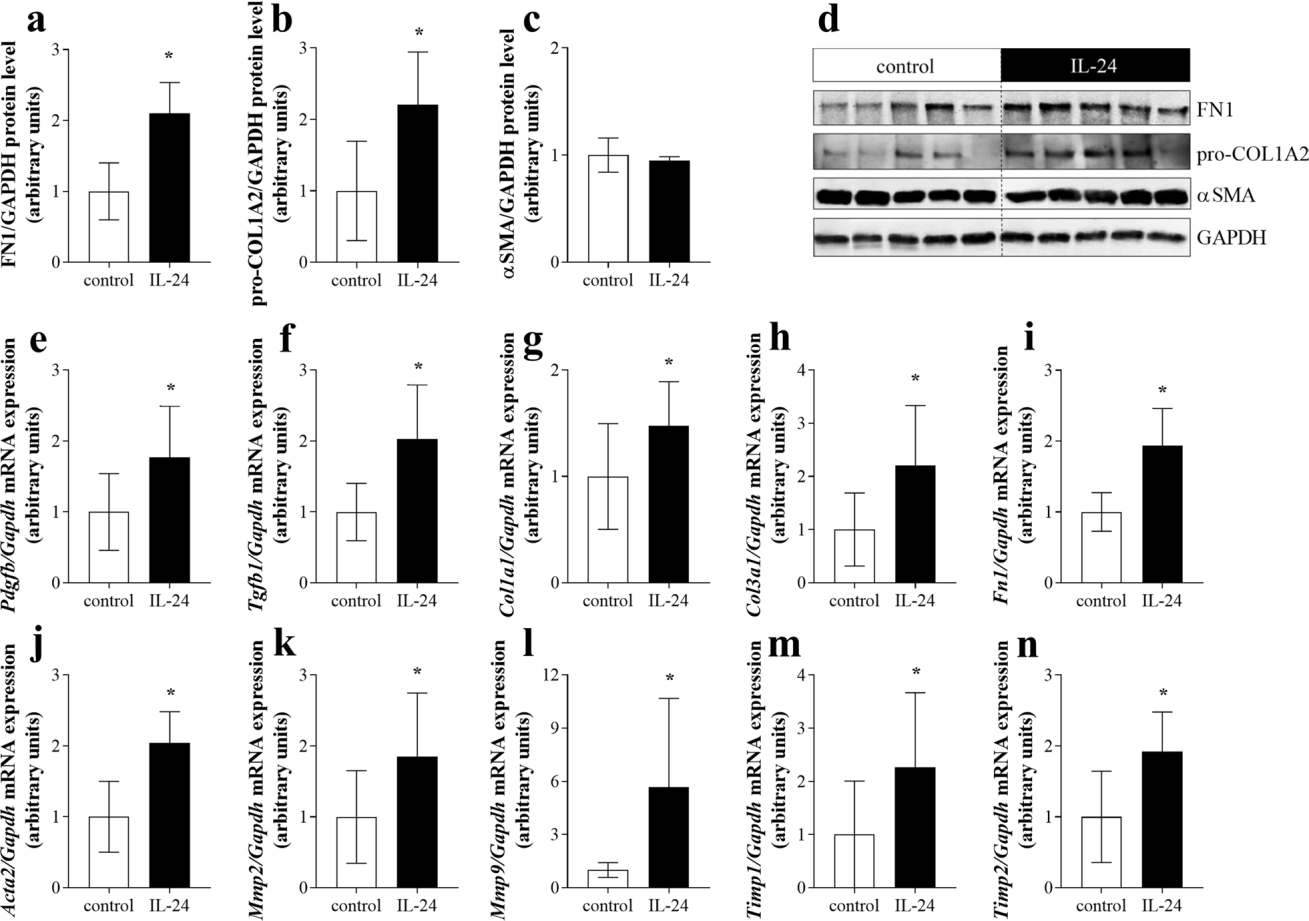
Tissue remodeling-associated factors in WT mice after IL-24 treatment. The protein level of fibronectin (FN1) (**a**, **d**), pro-collagen type I alpha 2 (pro-COL1A2) (**b**, **d**) and αSMA (**c**, **d**) and mRNA expression of *Tgfb1* (**e**), *Pdgfb* (**f**), *Col1a1* (**g**), *Col3a1* (**h**), *Fn1* (**i**), *Acta2* (j), *Mmp2* (**k**), *Mmp9* (**l**), *Timp1* (**m**) and *Timp2* (**n**) were determined by Western blot analysis and real-time PCR by comparison with GAPDH as internal control (n = 6). Results are presented as mean ± SD. *p < 0.05 vs. WT control (Mann–Whitney *U*-test)

In order to analyze the in vivo effect of IL24 on the remodeling of colon tissue, mice were treated locally with IL-24. Indeed, we found that the amount of FN1 and pro-COL1A2 increased in the colon of IL-24 treated mice compared to that of controls (Fig. 5a–d). Similarly, intracolonic injection of IL-24 increased the mRNA expression of *Tgfb1*, *Pdgfb*, *Col1a1*, *Col3a1*, *Fn1*, *Acta2*, *Mmp2*, *Mmp9*, *Timp1* and *Timp2* compared to that of vehicle treated mice (Fig. 5e–n).

### Body weight change and disease activity index of DSS-treated WT and *Il20rb* KO mice

**Fig. 6:**
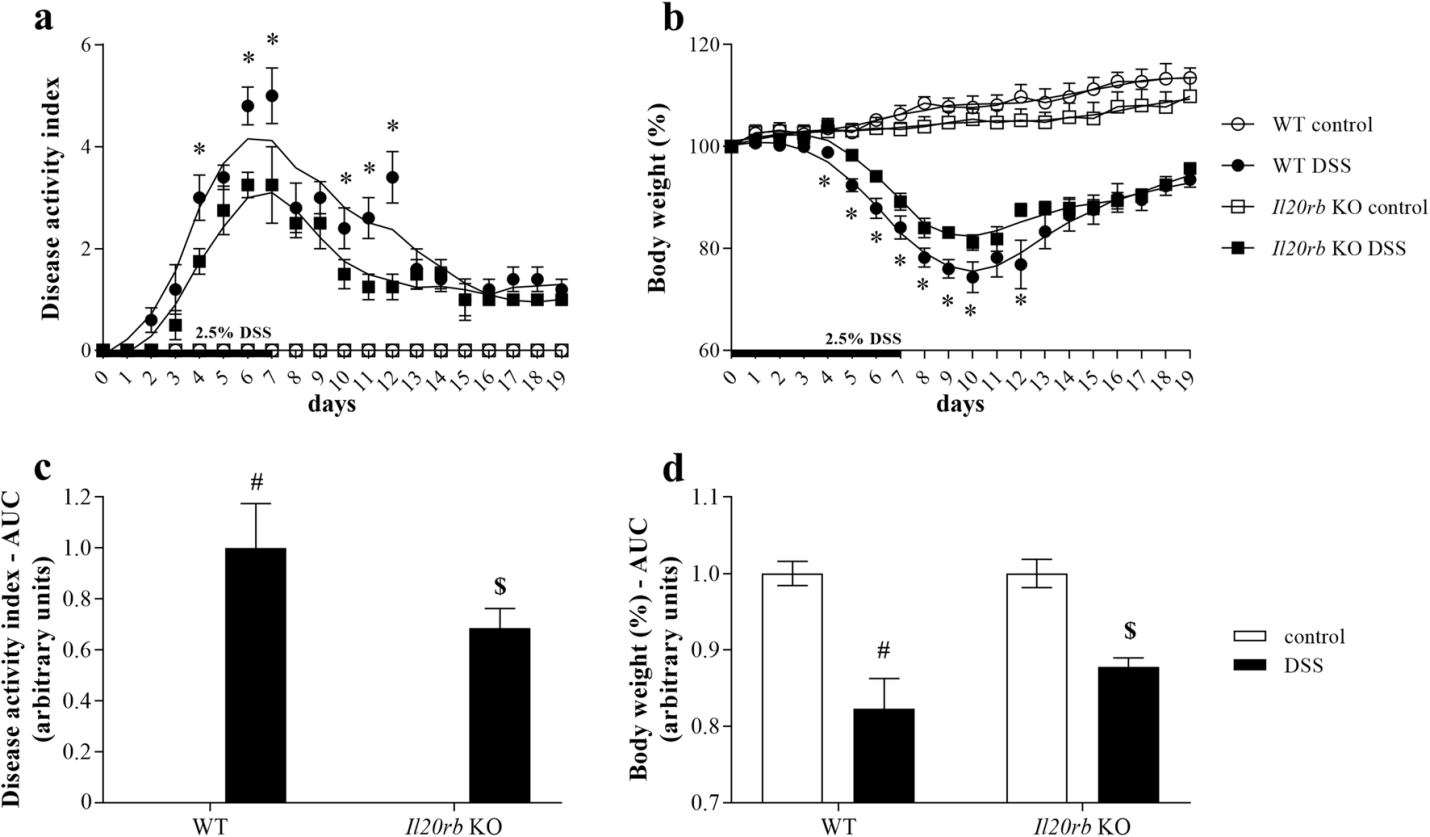
Severity of DSS-induced colitis in WT and *Il20rb* KO mice. Percentage of body weight change (**a**) and disease activity index of colitis (**b**) in DSS-treated mice (n = 6) was determined daily. Each point represents a mean value ± SEM. *p < 0.05 WT DSS vs. *Il20rb* KO DSS on the given day (Mann–Whitney *U*-test). Mean ± SD area under curve (AUC) values of body weight curves (**c**) and disease activity index (**d**). ^#^*p* < 0.05 vs. WT control; ^$^*p* < 0.05 vs. WT DSS (Mann–Whitney *U*-test)

Next, we investigated the consequences of IL-24 deficiency on the bodyweight and disease activity index of DSS treated WT and *Il20rb* KO mice. We found that DSS treatment resulted in decreased body weight (Fig. 6a, c) and increased DAI (Fig. 6b, d) of both WT and *Il20rb* KO mice. However, after induction of colitis the symptoms were significantly milder on several days and also cumulatively in the *Il20rb* KO group compared to WT mice.

### Tissue remodeling-associated factors in colon tissue of WT vs. *Il20rb* KO mice after DSS treatment

**Fig. 7:**
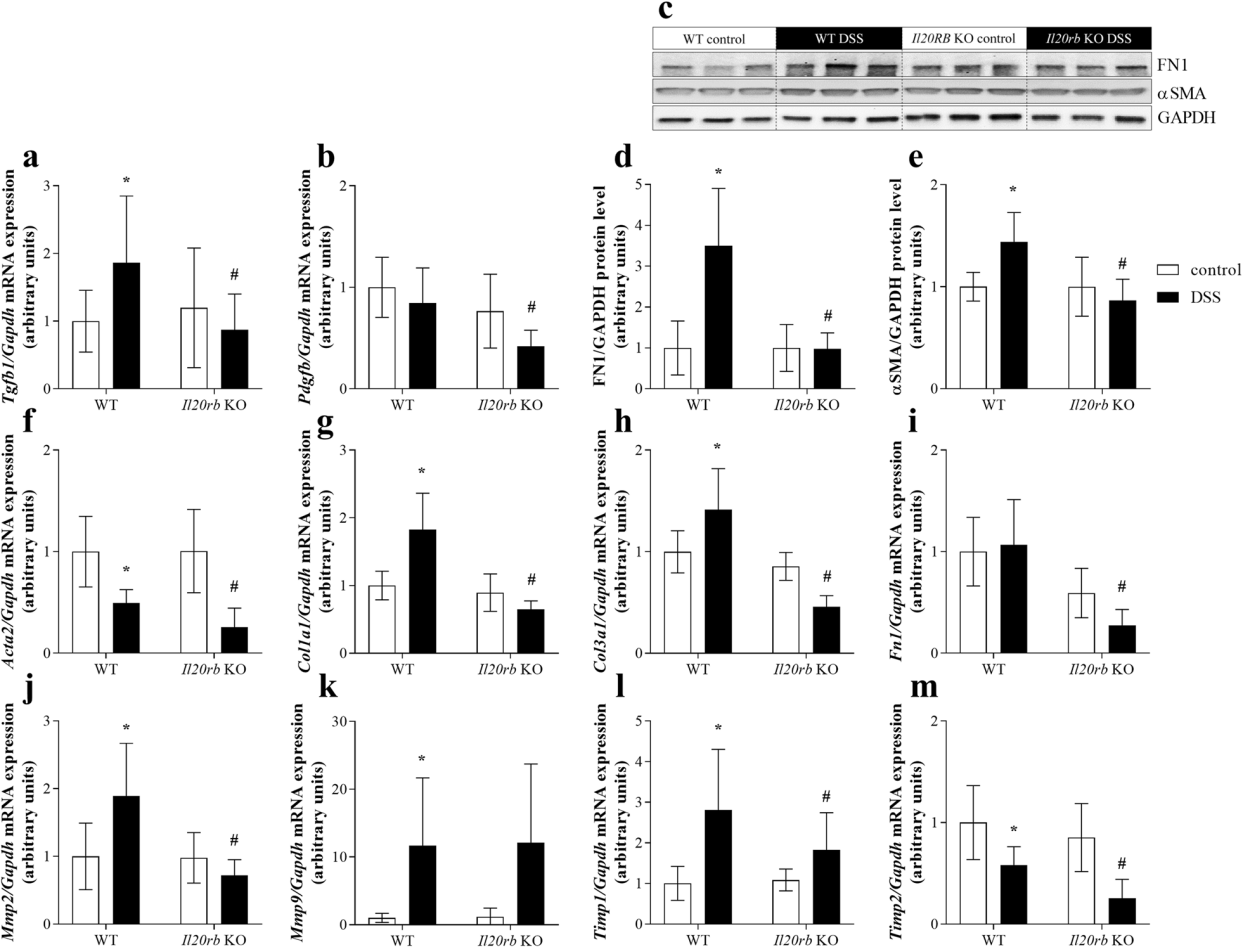
Tissue remodeling-associated factors in WT vs. *Il20rb* KO mice after DSS treatment. Relative protein level of FN1 (**c**, **d**) and αSMA (**c**, **e**) in colon tissue were measured by Western blot analysis, relative mRNA expression of *Tgfb1* (**a**), *Pdgfb* (**b**), *Col1a1* (**e**), *Col3a1* (**f**), *Fn1* (**g**), *Acta2* (**h**), *Mmp2* (**i**), *Mmp9* (**j**), *Timp1* (**k**) and *Timp2* (**l**) was measured by real-time PCR, by comparison with *Gapdh* as internal control (n = 6). Results are presented as mean ± SD. *p < 0.05 vs. WT control; ^#^*p* < 0.05 vs. WT DSS (Mann–Whitney *U*-test)

In the following measurements we investigated whether there is a difference in the expression of colonic tissue remodeling factors between DSS treated WT and *Il20rb* KO mice. DSS treatment of WT mice increased the colonic mRNA expression of *Tgfb1*, *Col1a1*, *Col3a1*, *Mmp2*, *Mmp9* and *Timp1* and decreased that of *Acta2* and *Timp2* compared to that of WT controls (Fig. 7a, b, f–m). Lack of IL-20RB resulted in less increased expression of *Tgfb1*, *Pdgfb*, *Col1a1*, *Col3a1*, *Fn1*, *Acta2*, *Mmp2*, *Timp1* and *Timp2* in the colon of DSS-treated KO than WT mice. Protein level of FN1 and αSMA was elevated in the colon of DSS treated WT mice compared to that of WT controls, but remained unchanged in the colon of *Il20rb* KO mice (Fig. 7c–e).

## Discussion

Maintenance the healthy architecture of the intestinal mucosa requires precise cooperation among immune system, epithelial cells and subepithelial MFs. In IBD the inappropriate, ongoing activation of immune response disturbs this fine-tuned process of mucosal remodeling leading to permanent activation of MFs and consequential excessive deposition of ECM resulting in malabsorption and motility disorders [6]. Indeed, approximately half of the IBD patients need one or more surgical intervention within their lifetime because of the scarring of the intestinal wall [5, 7].

Recently, increased amount of IL-24 has been reported in the mucosa of adult patients with IBD [19, 20]. It was demonstrated that IL-24 acts on colonic epithelial cells and elicits the activation of Janus kinase 1 (JAK-1)/STAT-3 dependent expression of membrane-bound mucins (MUC1, MUC3 and MUC4). Based on these observations Andoh et al. suggested that IL-24 may protect the integrity of inflamed mucosa of patients with IBD [19]. Nevertheless, our knowledge about the biological role of IL-24 in the inflamed intestinal mucosa is still insufficient. Therefore, in the present study we investigated the potential role of IL-24 on mucosal remodeling of therapy naive children with IBD.

Similarly, to previous studies of Andoh A. [19] and Fonseca-Camarillo G. et al. [20] we found increased amount of IL-24 in the inflamed colon mucosa of therapy naive children with CD or UC (Fig. 1) and also in that of DSS treated mice (Fig. 2).

To explore the underlying mechanisms responsible for the regulation of IL-24 synthesis we investigated the effect of different IBD-related factors, including IL-1β, IL-17, TNF-α, TGF-β, H_2_O_2_ and LPS [33] on the IL-24 expression in immune cells derived from peripheral blood (PBMCs) or from the lamina propria (LPMCs) (Fig. 1d–e). In these experiments, in line with previous results of us and others [21, 34], we found that PBMCs and also LPMCs are capable to produce IL-24 (Fig. 1). Indeed, similarly to recent studies [19] we found that IL-1ß acts as an inducer of IL-24 synthesis, however our data, in line with our previous results [18] demonstrated that LPS or H_2_O_2_ treatment has similar or even stronger effect on IL-24 synthesis in PBMCs and LPMCs. These data fit well into the literature suggesting the regulatory effect of LPS and IL-1ß on the IL-24 synthesis of immune cells [34].

It is more surprising that TNF-α, a well-known inflammatory factor, that induces the synthesis of several cytokines, decreased the synthesis of IL-24 by PBMCs (Fig. 1d). However, recently it has been shown that inhibition of the TNF-α induced NF-κB signaling pathway increases the synthesis of IL-24 in primary keratinocytes, suggesting that TNF-α itself may inhibit the synthesis of IL-24 [35]. Moreover, it is well known that in cells such as PBMCs or intestinal epithelial cells that frequently exposed to inflammatory triggers TNF-α rather inhibits the expression of different cytokines through ABIN3, IRAK-M, SOCS3 and ATF3 related pathways [36, 37]. Accordingly, previously we demonstrated that while TNF-α increase IL-24 synthesis by primary duodenal fibroblasts, it decrease that by FHs74Int, small intestinal epithelial cells [21]. Taken together our results suggest that TNF-α may represent a negative feedback mechanism inhibiting the overproduction of IL-24 in PBMCs.

Thereafter, we focused on understanding of the biological role of IL-24 in the pathomechanism of mucosal remodeling in IBD. We found that mucosal epithelial cells of children with IBD (Fig. 1) or those of DSS treated mice (Fig. 2) and also HT-29 colon epithelial cells (Fig. 3) express IL-20RB, the common IL-24 binding subunit of the IL-20RA/IL-20RB or IL-22RA/IL-20RB receptor heterodimers. Therefore, we investigated the effect of IL-24 on HT-29 colon epithelial cells. Previous studies demonstrated that IL-20 subfamily cytokines induce apoptosis of different cancer and non-cancerous epithelial cells [38–42], therefore we investigated the effect of IL-24 on cell viability. Surprisingly, in our experiments IL-24 treatment did not affect the viability of HT-29 colon epithelial cells (Additional file 2). More interestingly, similarly to our previous study on HK-2 renal tubular epithelial cells [18], we found that IL-24 induces HT-29 colon epithelial cells to synthesize TGF-β1 and PDGF-B (Fig. 3). Both growth factors play a well-known role in the regulation of tissue remodeling [33, 43]. Accordingly, TGF-ß1 treatment induced the expression of *COL1A1*, *COL3A1* and *FN1* by CCD-18Co colon fibroblasts (Additional file 5) leading to the massive ECM deposition as demonstrated by SiriusRed assay. Similarly, PDGF-B increased the proliferation and *COL1A1* and *COL3A1* expression by CCD-18Co colon fibroblasts (Additional file 5).

Since CCD-18Co colon fibroblasts also showed IL-20RB immunopositivity (Fig. 4), we investigated the direct effects of IL-24 on these cells, as well. We found that IL-24 treatment affects neither the viability nor the proliferation or the ECM production by colon fibroblasts, even if they were co-treated with TGF-ß1 or PDGF-B (Additional file 4).

However, IL-24 treatment enhanced the cell-free gap closure ability of colon fibroblasts (Fig. 4), which in light of the fact that IL-24 did not enhance the proliferation of the cells (Additional file 4), suggest that it increased their motility. In line with this observation we found that IL-24 treatment increased the expression of gelatinases, including matrix metallopeptidases *MMP2* and *MMP9,* and also the expression of their natural inhibitors, the tissue inhibitors of metalloproteinases *TIMP1* and *TIMP2* (Fig. 4). It is known, that MMP-2 and MMP-9 are abundantly expressed in the mucosa of patients with IBD and their expression highly correlates with the disease activity [44, 45]. Indeed, MMPs are responsible for not only the degradation of ECM, but also for increased proliferation and migration of various cells [46, 47]. Moreover, due to their proteolytic activity MMPs may also facilitate the cleavage of the inactive form of inflammatory factors, including IL-1β or TNF-α, thus facilitating local inflammation [48]. Similarly, TIMPs are also involved in the proliferation and migration of different cells as well [49–52]. Taking together, our present in vitro results suggest that IL-24 may regulate the mucosal remodeling through the enhanced production of TGF-β and PDGF-B of colon epithelial cells, and also through its effects on the migration of subepithelial MFs.

In a next step we investigated whether IL-24 may influence the synthesis of tissue remodeling related factors in vivo*,* as well. We found that local IL-24 treatment of the colon, similarly to our in vitro findings, increased the amount of FN1 and pro-COL1A2, and also the expression of the core pro-fibrotic factors including *Tgfb1*, *Pdgfb* and that of *Col1a1*, *Col3a1*, *Fn1*, *Acta2*, *Mmp2*, *Mmp9*, *Timp1* and *Timp2* (Fig. 5). Although these data demonstrate the potential role of IL-24 in the remodeling of the normal non inflamed colon, it does not elucidate the role of IL-24 in the inflamed mucosa of patients with IBD. Therefore, in the next experiment we investigated the significance of IL-24 also in the inflamed colon of DSS treated WT and *Il20rb* KO [27] mice. In this in vivo experiment the acute inflammation was followed by a regeneration phase ensuring mucosal remodeling and fibrosis [53–55]. We found that DSS treatment induced severe loss of body weight, diarrhea, hematochezia and deteriorating general condition with abdominal pain in both WT and *Il20rb* KO mice. However, the symptoms of colitis were significantly milder in *Il20rb* KO mice suggesting that activity of cytokines of IL-20 subfamily may worsen the symptoms of colitis (Fig. 6). Similarly, we found that the expression of pro-fibrotic, tissue remodeling related factors, including *Tgfb1*, *Pdgfb*, *Col1a1*, *Col3a1*, *Fn1*, *Acta2*, *Mmp2*, *Timp1* and *Timp2*, were increased to a lesser extent in the colon of *Il20rb* KO compared to that of WT mice (Fig. 7). These results are in accordance with our previous study demonstrating decreased ECM deposition in the kidney of *Il20rb* KO mice underwent unilateral ureteral obstruction compared to that of WT animals [18]. Taken together our above in vivo experiment demonstrate that IL-20 cytokines have strong and multiple effects on tissue remodeling of the inflamed colon. Although the effects of IL-19, IL-20 and IL-24 are also altered in *Il20rb* KO mice, we believe that the role of IL-24 is prominent. Our hypothesis is supported by the in vivo and in vitro experiments of the present study demonstrating the direct effect of IL-24 treatment on TGF-β and PDGF-B synthesis. Moreover, similarly to our results recently, Rao et al. demonstrated that the lack of IL-24 resulted in decreased amount of TGF-ß and ECM in the lung of bleomycin treated *Il24* KO mice compared to that of WT animals [16].

## Conclusions

In summary, although there are limitations of our study, mainly the relatively small number of the enrolled therapy naive children with IBD, which do not allow to compare the amount of IL-24 and severity of clinical symptoms, we made significant progress in the understanding of biological effects of IL-24 in the pathomechanism of IBD. Indeed, increased amount of IL-24 was demonstrated in the mucosa of therapy naive children with IBD. Moreover, our in vivo experiments demonstrated that activation of IL-20RB significantly influence the severity of colitis. Our experiments also revealed that IL-24 treatment induces the synthesis of TGF-β1 and PDGF-B by colonic epithelial cells in vitro and by colonic mucosa in vivo. We demonstrated that IL-24 enhances the migratory capacity of CCD-18Co cells suggesting their increased activation. Similarly, we demonstrated that IL-24 enhances the expression of MMP-2 and-9 and also TIMP-1 and -2 of colon fibroblasts and also that of the colon in vivo.

**Fig. 8:**
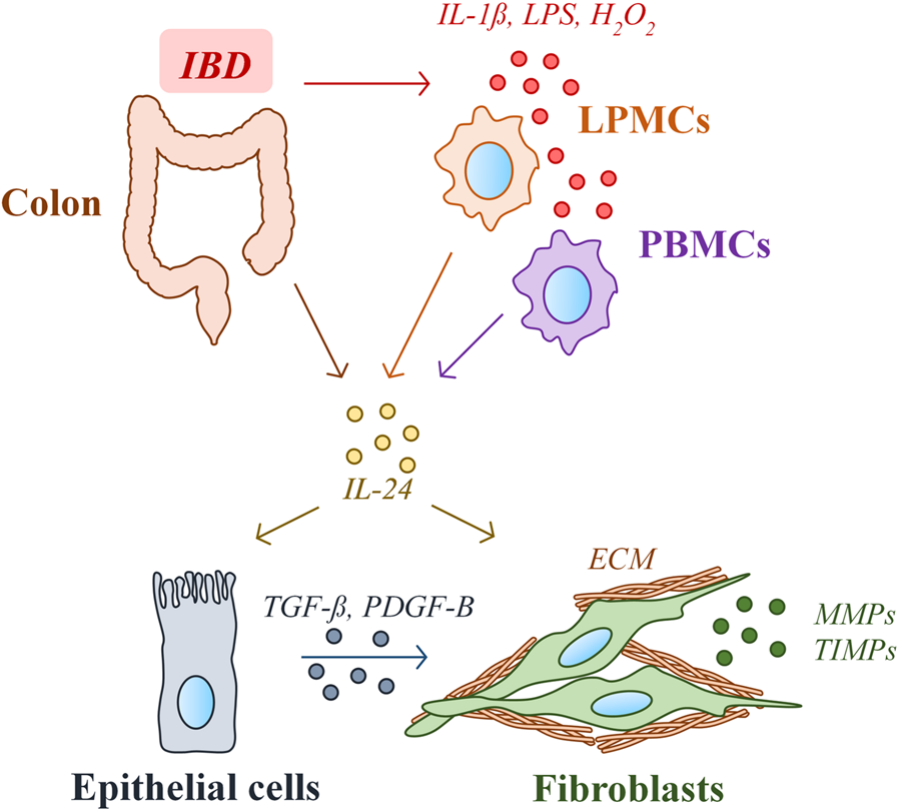
Schematic model of the proposed mechanism underlying the role of IL-24 in the IBD-associated tissue remodeling. Our results showed increased amount of IL-24 in the inflamed colonic mucosa of IBD patients or DSS treated mice. IBD-related factors, including IL-1ß, LPS or H_2_O_2_ are direct inducers of IL-24 in immune cells derived from lamina propria (LPMCs) or peripheral blood (PBMCs). IL-24 enhances the synthesis of pro-fibrotic factors, including TGF-β and PDGF-B in epithelial cells. These growth factors together with IL-24 induce the activation of colonic fibroblast leading to the excessive deposition of extracellular matrix (ECM) components

Taken together our data suggest that IL-24 may play a central role in the IBD-associated tissue remodeling (Fig. 8). Currently, anti-TNF drugs play a significant role in the therapy of IBD, however a number of patients lose sensitivity or do not respond to the therapy at all [56, 57]. Therefore, the better understanding of molecular processes of IBD may contribute to the identification of novel biomarkers or to the development of new drugs that can complete the current therapeutic options to treat IBD.

## Supplementary Information

**Additional file 1**. Nucleotide sequences of primer pairs, product length and specific annealing temperatures applied for the real-time reverse transcriptase polymerase chain reaction (RT- PCR) detection. *F* forward, *R* reverse, *bp* base pair, *T*_*a*_ annealing temperature.

**Additional file 2**. Effect of IL-24 on the viability of HT-29 colon epithelial cells. Rate of apoptotic cells was measured by Annexin V assay (n = 6). Results are presented as percentage of total cells, mean ± SD.

**Additional file 3**. Effect of IL-24 on the viability of CCD-18Co colon fibroblast cells. Rate of apoptotic cells was measured by Annexin V assay (n = 6). Results are presented as percentage of total cells, mean ± SD.

**Additional file 4**. Effect of IL-24 on proliferation and collagen deposition of CCD-18Co cells. Cell proliferation (**a**) was investigated by MTT assay in the absence or presence of PDGF-B treatment (m = 5). Collagen deposition (**b**) was measured by SiriusRed assay in the absence or presence of TGF-ß treatment (n = 5). Cytotoxic effect of the applied treatments was monitoring by LDH assays (n = 5). Results are presented in percentage of untreated group (0 ng/ml IL-24 control) as mean** ± **SD. *p < 0.05 vs. *control at 0 ng/ml IL-24* (multiple *t*-test), ^#^*p* < 0.05 vs. *PDGF-B/TGF-ß at 0 ng/ml IL-24* (two-way ANOVA).

**Additional file 5**. Effect of IL-24, TGF-ß and PDGF-B on the ECM production of CCD-18Co colon fibroblast cells. After treatment with IL-24 (**a**), TGF-ß (**b**) or PDGF-B (**c**), relative mRNA expressions of *COL1A1*, *COL3A1* and *FN1* in CCD-18Co colon fibroblast cells were measured by real-time PCR, by comparison with *GAPDH* as internal control (n = 6). Results are presented as mean ± SD. *p < 0.05 vs. control (Mann–Whitney *U*-test).

## Data Availability

The datasets used and/or analyzed during the current study are available from the corresponding author on reasonable request.

## Abbreviations

AUC: Area under curve
CD: Crohn’s disease
DAI: Disease activity index
DSS: Dextran sodium sulphate
ECM: Extracellular matrix
FBS: Fetal bovine serum
FN1: Fibronectin
IBD: Inflammatory bowel disease
IL-: Interleukin
KO: Knock out
LDH: Lactate dehydrogenase
LPS: Lipopolysaccharide
MFI: Mean fluorescence intensity
MFs: Myofibroblasts
MMPs: Matrix metalloproteases
PBMC: Peripheral mononuclear blood cell
PBS: Phosphate buffered saline
PCDAI: Pediatric Crohn’s Disease Activity Index
PCUAI: Pediatric Ulcerative Colitis Activity Index
RT: Room temperature
TBS: TRIS-buffered saline
TIMPs: Tissue inhibitors of metalloproteinases
UC: Ulcerative colitis
WT: Wild type
αSMA: Smooth muscle actin α
