# Supplementary material for: Interleukin-24 regulates mucosal remodeling in inflammatory bowel diseases
Source: J Transl Med. 2021 Jun 2;19:237. doi: 10.1186/s12967-021-02890-7 (PMC8173892; doi:10.1186/s12967-021-02890-7)
Supplement: Supplementary file 1 — Additional file 1. Nucleotide sequences of primer pairs, product length and specific annealing temperatures applied for the real-time reverse transcriptase polymerase chain reaction (RT- PCR) detection. F forward, R reverse, bp base pair, Ta annealing temperature. [file 12967_2021_2890_MOESM1_ESM.docx]

**Additional file 1.** Nucleotide sequences of primer pairs, product length and specific annealing temperatures applied for the real-time reverse transcriptase polymerase chain reaction (RT- PCR) detection. F: forward; R: reverse; bp: base pair; T_a_: annealing temperature

| **Species** | **Gene** | **Primer pairs** | | **Product lenght** | **T_a_** |
| --- | --- | --- | --- | --- | --- |
| human | *COL1A1* | F: | 5'-CTG CCC CGC CGC CGA AGT C-3' | 96 bp | 63 °C |
|  |  | R: | 5'-CCC TCG ACG CCG GTG GTT TCT TG-3' |  |  |
| human | *COL3A1* | F: | 5'-TGC CCA CAG CCT CCA ACT-3' | 113 bp | 56 °C |
|  |  | R: | 5'-ATA CCA GGG TCA CCA TTT CTC C-3' |  |  |
| human | *FN1* | F: | 5'-GGC TGC CCA CGA GGA AAT CTG C-3' | 229 bp | 56 °C |
|  |  | R: | 5'-GTG CCC CTC TTC ATG ACG CTT GTG-3' |  |  |
| human | *GAPDH* | F: | 5'-AGC AAT GCC TCC TGC ACC ACC AA-3' | 159 bp | 60 °C |
|  |  | R: | 5'-GCG GCC ATC ACG CCA CAG TTT-3' |  |  |
| human | *IL24* | F: | 5'-AGG CGG TTT CTG CTA TTC C-3' | 55 bp | 48 °C |
|  |  | R: | 5'-GAG CTG CTT CTA CGT CCA ACT-3' |  |  |
| human | *IL20RB* | F: | 5'-GGT GCC CAG GAA CAT GTC AAA AT-3' | 188 bp | 58 °C |
|  |  | R: | 5'-AGG GCC AGT ACC AGG GGA ATG-3' |  |  |
| human | *MMP2* | F: | 5'-CAC GCT GGG CCC TGT CAC TCC T-3' | 205 bp | 60 °C |
|  |  | R: | 5'-TGG GGC CTC GTA TAC CGC ATC AAT-3' |  |  |
| human | *MMP9* | F: | 5'-CGC GGG CGG TGA TTG ACG AC-3' | 198 bp | 63 °C |
|  |  | R: | 5'-GAA TGC CGG GGC CAG GAG GAA-3' |  |  |
| human | *TIMP1* | F: | 5'-ACC CCC GCC ATG GAG AGT G-3' | 223 bp | 58 °C |
|  |  | R: | 5'-AGG GGA TGG ATA AAC AGG GAA ACA-3' |  |  |
| human | *TIMP2* | F: | 5'-CAG GGC CAA AGC GGT CAG TGA GA-3' | 151 bp | 57 °C |
|  |  | R: | 5'-CGA GGA GGG GGC CGT GTA GAT AAA CT-3' |  |  |
| mouse | *Acta2 (1)* | F: | 5'-CCC CTG AAG AGC ATC GGA CA-3' | 105 bp | 60 °C |
|  |  | R: | 5'-TGG CGG GGA CAT TGA AGG T-3' |  |  |
| mouse | *Col1a1* | F: | 5'-CAA AGC TGC TGA TGG TTC T-3' | 107 bp | 60 °C |
|  |  | R: | 5'-GAC CAG CTT CAC CCT TG-3' |  |  |
| mouse | *Col3a1* | F: | 5'-GTC CCC TGG CTC AAA TGG CTC AC-3' | 175 bp | 61 °C |
|  |  | R: | 5'-GGG GCC CCT TGC TCC TAT TAG TCC-3' |  |  |
| mouse | *Fn1* | F: | 5'-GGT CAG GGC CGG GGC AGA T-3' | 228 bp | 60 °C |
|  |  | R: | 5'-CTG GCT GGG GGT CTC CGT GAT AAT-3' |  |  |
| mouse | *Gapdh* | F: | 5'-ATC TGA CGT GCC GCC TGG AGA AAC-3' | 164 bp | 60 °C |
|  |  | R: | 5'-CCC GGC ATC GAA GGT GGA AGA GT-3' |  |  |
| mouse | *Il24* | F: | 5'-TGT GGG AGG CCT TCT GGA CTG T-3' | 133 bp | 57 °C |
|  |  | R: | 5'-AGC AGG CTG TGG GCA AGG TAA C-3' |  |  |
| mouse | *Mmp2* | F: | 5'-ACC ACC GAG GAC TAT GAC-3' | 121 bp | 60 °C |
|  |  | R: | 5'-TGT TGC CCA GGA AAG TGA-3' |  |  |
| mouse | *Mmp9* | F: | 5'-TGC CCT AGT GAG AGA CTC TAC A-3' | 128 bp | 60 °C |
|  |  | R: | 5'-CAG CGG TAA CCA TCC GA-3' |  |  |
| mouse | *Pdgfb* | F: | 5'-CTG GGC GCT CTT CCT TCC TCT C-3' | 170 bp | 60 °C |
|  |  | R: | 5'-CCA GCT CAG CCC CAT CTT CAT C-3' |  |  |
| mouse | *Tgfb1* | F: | 5'-GTG CGG CAG CTG TAC ATT GAC TTT-3' | 239 bp | 60 °C |
|  |  | R: | 5'-GGC TTG CGA CCC ACG TAG TAG AC-3' |  |  |
| mouse | *Timp1* | F: | 5'-CAC GGG CCG CCT AAG GAA C-3' | 249 bp | 58 °C |
|  |  | R: | 5'-CGT GGC AGG CAA GCA AAG TG-3' |  |  |
| mouse | *Timp2* | F: | 5'-TGC CCT GGC ACA CGC TTA GC-3' | 221 bp | 58 °C |
|  |  | R: | 5'-GTA CCA CGC GCA AGA ACC ATC A-3' |  |  |

Reference

1. Veres-Szekely A, Pap D, Sziksz E, Javorszky E, Rokonay R, Lippai R, et al. Selective measurement of a smooth muscle actin: why beta-actin can not be used as a housekeeping gene when tissue fibrosis occurs. BMC Mol Biol. 2017;18.
