# Supplementary figures and images for: Interleukin-24 regulates mucosal remodeling in inflammatory bowel diseases
Source: J Transl Med. 2021 Jun 2;19:237. doi: 10.1186/s12967-021-02890-7 (PMC8173892; doi:10.1186/s12967-021-02890-7)

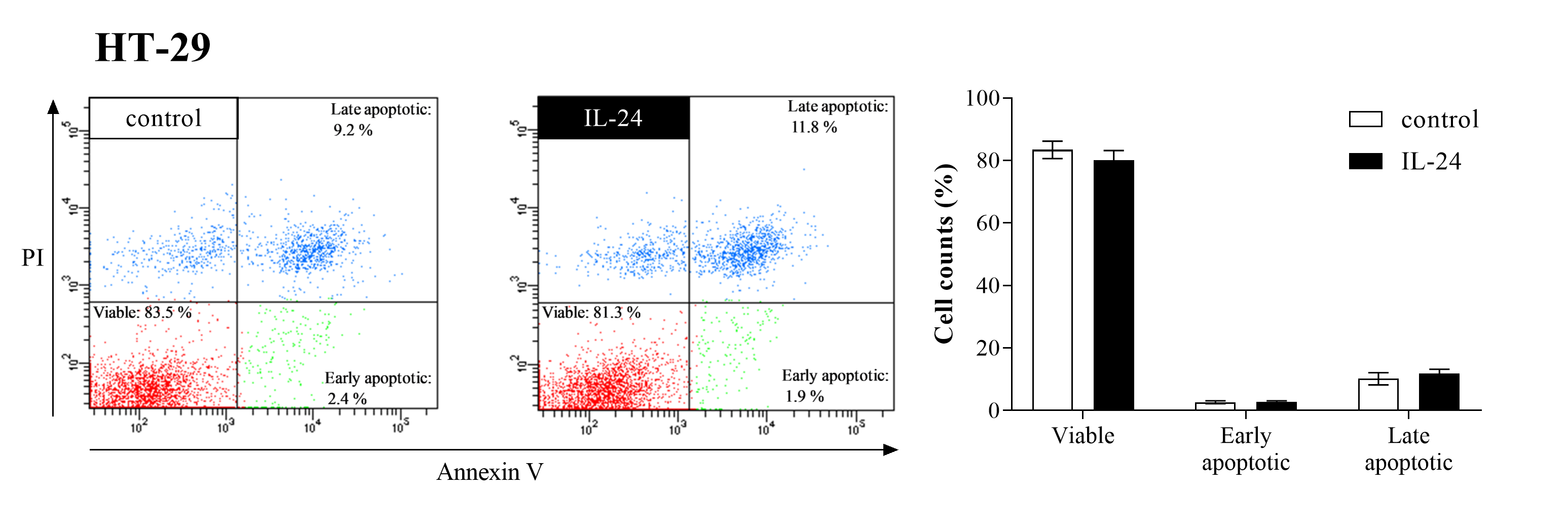

Supplement: Supplementary file 2 — Additional file 2. Effect of IL-24 on the viability of HT-29 colon epithelial cells. Rate of apoptotic cells was measured by Annexin V assay (n = 6). Results are presented as percentage of total cells, mean ± SD. [file 12967_2021_2890_MOESM2_ESM.tif]

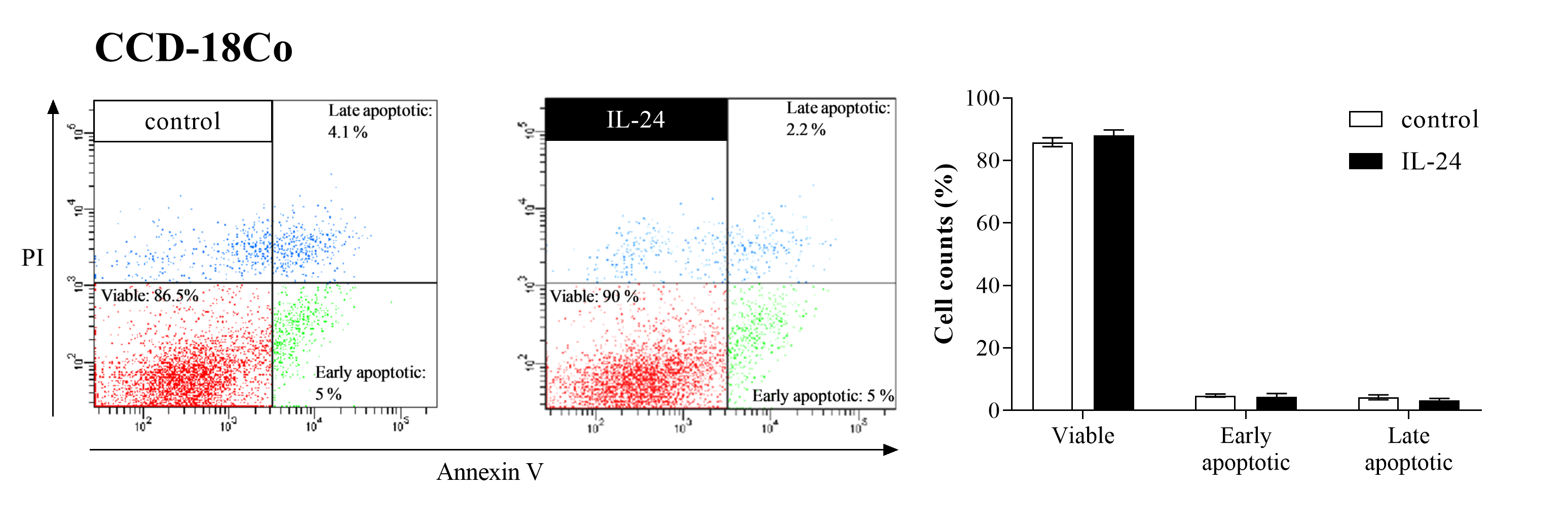

Supplement: Supplementary file 3 — Additional file 3. Effect of IL-24 on the viability of CCD-18Co colon fibroblast cells. Rate of apoptotic cells was measured by Annexin V assay (n = 6). Results are presented as percentage of total cells, mean ± SD. [file 12967_2021_2890_MOESM3_ESM.tif]

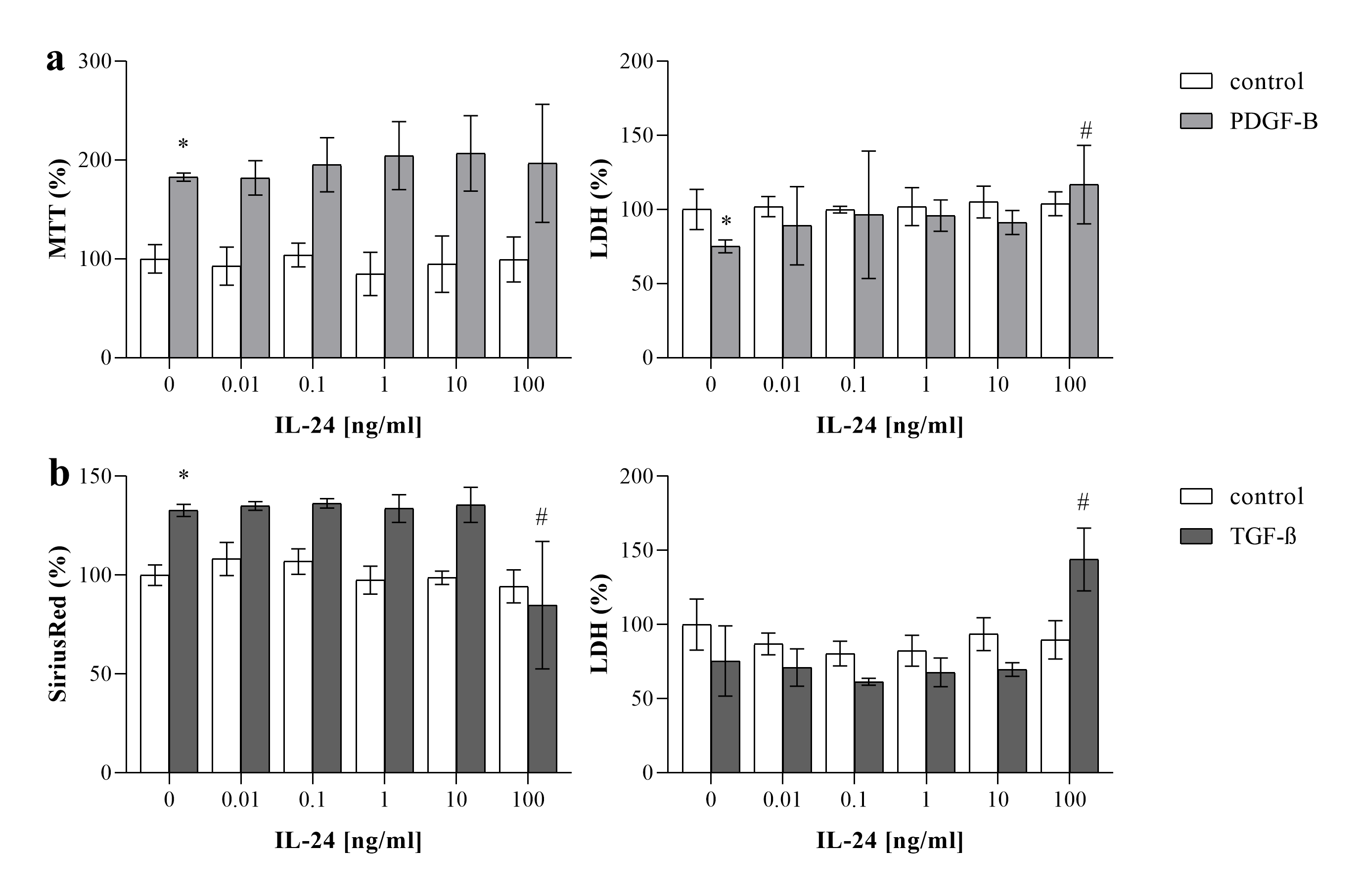

Supplement: Supplementary file 4 — Additional file 4. Effect of IL-24 on proliferation and collagen deposition of CCD-18Co cells. Cell proliferation (a) was investigated by MTT assay in the absence or presence of PDGF-B treatment (m = 5). Collagen deposition (b) was measured by SiriusRed assay in the absence or presence of TGF-ß treatment (n = 5). Cytotoxic effect of the applied treatments was monitoring by LDH assays (n = 5). Results are presented in percentage of untreated group (0 ng/ml IL-24 control) as mean ± SD. *p < 0.05 vs. control at 0 ng/ml IL-24 (multiple t-test), #p < 0.05 vs. PDGF-B/TGF-ß at 0 ng/ml IL-24 (two-way ANOVA). [file 12967_2021_2890_MOESM4_ESM.tif]

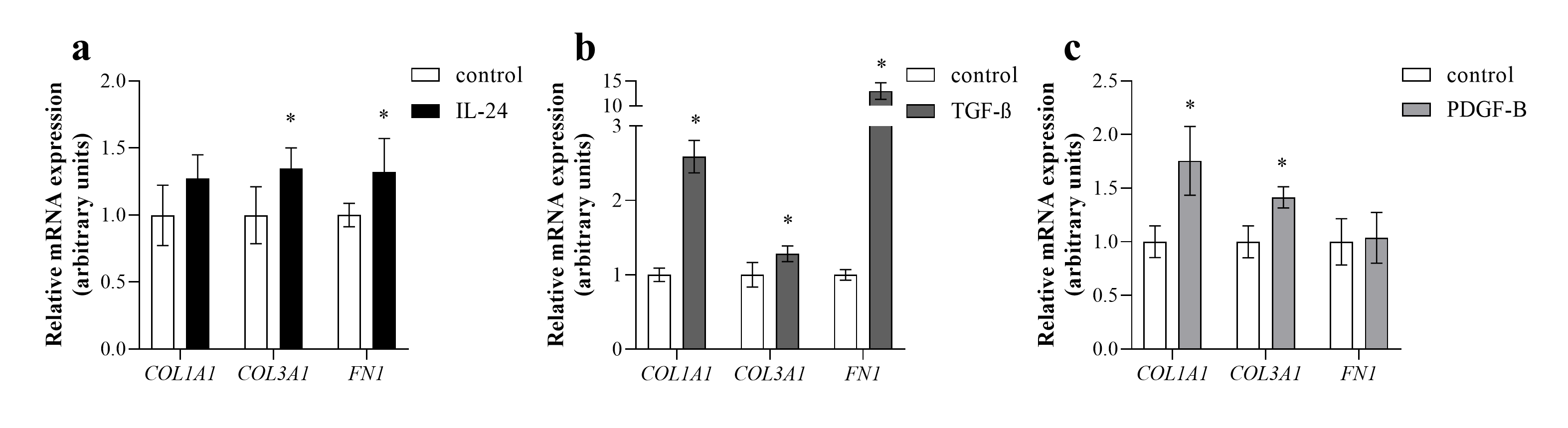

Supplement: Supplementary file 5 — Additional file 5. Effect of IL-24, TGF-ß and PDGF-B on the ECM production of CCD-18Co colon fibroblast cells. After treatment with IL-24 (a), TGF-ß (b) or PDGF-B (c), relative mRNA expressions of COL1A1, COL3A1 and FN1 in CCD-18Co colon fibroblast cells were measured by real-time PCR, by comparison with GAPDH as internal control (n = 6). Results are presented as mean ± SD. *p < 0.05 vs. control (Mann–Whitney U-test). [file 12967_2021_2890_MOESM5_ESM.tif]
